# Exercise Is More Effective at Altering Gut Microbial Composition and Producing Stable Changes in Lean Mass in Juvenile versus Adult Male F344 Rats

**DOI:** 10.1371/journal.pone.0125889

**Published:** 2015-05-27

**Authors:** Agnieszka Mika, Will Van Treuren, Antonio González, Jonathan J. Herrera, Rob Knight, Monika Fleshner

**Affiliations:** 1 Department of Integrative Physiology and the Center for Neuroscience, University of Colorado, Boulder, Colorado, 80301, United States of America; 2 Department of Microbiology and Immunology, Stanford University, Stanford, California, 94305, United States of America; 3 Departments of Pediatrics, University of California San Diego, La Jolla, California, 29093, United States of America; 4 Computer Science & Engineering, University of California San Diego, La Jolla, California, 29093, United States of America; Teagasc Food Research Centre, IRELAND

## Abstract

The mammalian intestine harbors a complex microbial ecosystem that influences many aspects of host physiology. Exposure to specific microbes early in development affects host metabolism, immune function, and behavior across the lifespan. Just as the physiology of the developing organism undergoes a period of plasticity, the developing microbial ecosystem is characterized by instability and may also be more sensitive to change. Early life thus presents a window of opportunity for manipulations that produce adaptive changes in microbial composition. Recent insights have revealed that increasing physical activity can increase the abundance of beneficial microbial species. We therefore investigated whether six weeks of wheel running initiated in the juvenile period (postnatal day 24) would produce more robust and stable changes in microbial communities versus exercise initiated in adulthood (postnatal day 70) in male F344 rats. 16S rRNA gene sequencing was used to characterize the microbial composition of juvenile versus adult runners and their sedentary counterparts across multiple time points during exercise and following exercise cessation. Alpha diversity measures revealed that the microbial communities of young runners were less even and diverse, a community structure that reflects volatility and malleability. Juvenile onset exercise altered several phyla and, notably, increased Bacteroidetes and decreased Firmicutes, a configuration associated with leanness. At the genus level of taxonomy, exercise altered more genera in juveniles than in the adults and produced patterns associated with adaptive metabolic consequences. Given the potential of these changes to contribute to a lean phenotype, we examined body composition in juvenile versus adult runners. Interestingly, exercise produced persistent increases in lean body mass in juvenile but not adult runners. Taken together, these results indicate that the impact of exercise on gut microbiota composition as well as body composition may depend on the developmental stage during which exercise is initiated.

## Introduction

The mammalian gut contains an estimated 100 trillion commensal microorganisms that have collectively co-evolved to enrich host physiology [1]. A large body of work decisively demonstrates that these microorganisms are critical for the development and function of many physiological systems. Studies comparing germ-free mice (GF mice; mice bred in sterile conditions and lacking gut microbiota) to conventional mice have revealed that bacterial colonization of the intestine is important for nutrient synthesis and uptake [2–4], immune regulation/tolerance [5,6], the development of primary and secondary lymphoid tissues [7,8], and an intact gastrointestinal barrier [9].

In addition to the importance of an intact gut microbiota, the overall phylogenetic composition and the presence of specific species can confer benefits on host health. At the phylum level, there is evidence by some [10–13] but not all [14,15] researchers that an increased Bacteroidetes to Firmicutes ratio is linked to a lean phenotype [10–13] and increases in the production of short-chain fatty acids (SCFAs) that promote energy expenditure. At the species level, some *Bacteroides spp*. [16] and indigenous *Clostridium spp*. [17] can facilitate T regulatory cell differentiation and induce anti-inflammatory immune responses. Recently, evidence has revealed that specific microorganisms can even influence brain plasticity and emotional behavior. For example, *Bifidobacteria* and *Lactobacillus spp*. can attenuate anxiety and depressive-like behavior in rodents [18,19], as well as humans [20]. Thus, given that specific microorganisms at both the phylum and species levels can promote aspects of host health, the microbial composition of the intact gut is important to consider.

Interestingly, recent research demonstrates that the impact of the gut microbiota on host physiology can be age dependent. Studies using GF mouse models have revealed an early sensitive period during which the absence of an intact gut microbiota results in irreversible behavioral and physiological consequences. For instance, exaggerated HPA-responses exhibited by GF mice can only be partially normalized with *Bifidobacteria infantis* if given in early life [21]. Similarly, GF mice exhibit altered anxiety behavior in the elevated plus-maze that can be normalized by exposure to microbial populations from conventionally raised mice, but not if exposure occurs in adulthood [22]. Early exposure to certain microorganisms can also program the immune system. For example, inoculation with *Bifidobacteria spp*. produced oral tolerance in GF mice only if administered early in development [23]. In addition to evidence from GF models, antibiotic-induced perturbations in microbiota during early, critical periods can create lasting changes in host physiology. Indeed, it was recently demonstrated that antibiotic administration in early life produced increases in total body mass as well as fat mass in adulthood [24]. Evidence from humans also suggests that bacterial community structure and composition during development can have long-lasting phenotypic impacts. For example, one study in humans revealed that infant microbial composition was predictive of childhood obesity [25], while others have shown that early life antibiotic administration is associated with increased adiposity [26,27]. These findings reveal that the presence and composition of the gut microbiota during development can program host health throughout the lifespan.

Physiological systems of the developing organism are highly malleable and sensitive to change, and the gut microbial community is similarly more plastic and volatile early in life [28–32]. To our knowledge, the most comprehensive study to date investigating microbial diversity across age included samples from infants, children, adolescents, and adults and demonstrated that interpersonal variation in microbial composition was significantly greater in children versus adults [30]. Importantly, this work also revealed that bacterial diversity increased across age. It has been suggested that the increased stability and complexity of the adult microbiota provides resistance against long-term changes in composition [28]. The early life microbial ecosystem may therefore be more sensitive to environmental change because it is less stable and diverse than the adult microbiota. Thus, environmental manipulations that produce adaptive changes in community structure could potentially have a greater and more lasting impact on the microbiota if implemented in early life.

Exercise is one such environmental manipulation capable of changing gut microbial composition in a manner that could potentially benefit the host. For instance, six days of wheel running exercise increased *Bifidobacterium* and *Lactobacillus spp*. [33], species implicated in mood and lean body composition [34]. Another group reported that wheel running significantly altered overall microbial composition and increased n-butyrate concentrations [35]. This particular SCFA is capable of increasing host energy expenditure [36]. Notably, in another study, wheel running prevented high-fat diet associated weight gain and produced a microbial composition similar to lean mice [37]. Although some evidence suggests that exercise can adaptively alter gut microbial composition, no research to date has considered the developmental stage of exercise initiation, nor investigated the stability of these exercise-induced changes across the lifespan. Given the state of plasticity of the developing gut and the therapeutic potential of manipulating bacterial composition in early life, it is important to investigate whether exercise-induced changes in composition are greater and more stable if exercise is initiated earlier in development. Furthermore, given the relationship between exercise-altered microbiota and metabolic adaptations, it is possible that early life exercise could produce microbial patterns that promote leanness as well as lasting, beneficial changes in body composition.

To explore this, adult, postnatal day (PND) 70 and juvenile, PND 24, male F344 rats were housed in standard cages with or without running wheels for six weeks. Wheel running in rodents is rewarding [38] and produces a multitude of beneficial effects including increased endurance [39], decreased visceral adiposity [40], and increased stress robustness [41–44]. Additionally, wheel running is a natural behavior as rodents in the wild will choose to run on wheels if given access [45].

In the present study, gut microbial composition was assessed using 16S rRNA gene sequencing in fecal samples from juvenile and adult runners and their sedentary counterparts. Samples were collected after three days and six weeks of wheel running, and 25 days after running had stopped. 16S rRNA gene sequencing provides a comprehensive measure of microbial ecology without the bias of traditional culture methods. To assess possible physiological consequences associated with an early life exercise-altered microbial configuration, long lasting adaptations in body composition were also investigated in separate cohorts of rats using chemical carcass analysis. We hypothesize that exercise initiated during the juvenile period will produce more robust and stable adaptations in gut microbial composition than exercise initiated in adulthood, and that these changes will be associated with a lean phenotype.

## Materials and Methods

### Subjects and Housing

Juvenile, PND 24 (postnatal day; n = 20) and adult, PND 70 (n = 20), male Fischer F344 rats (Harlan Laboratories, IN) were housed in a temperature (22°C) and humidity controlled environment and maintained on a 12:12-hr light: dark cycle, and fed a standard diet containing 18.6% protein, 6% fat and 3.5% fiber (Harlan Laboratories, IN). All rats were pair housed in Nalgene Plexiglas cages (45 x 25.2 x 14.7 cm). Pair housing was necessary in these experiments due to the stressful nature of single housing juveniles [46]. Care was taken to minimize discomfort during all procedures, and all experimental protocols were approved by the University of Colorado Animal Care and Use Committee. All rats were weighed weekly, and had *ad libitum* access to food and water.

### Voluntary exercise

Immediately upon arrival, juvenile and adult rats were randomly assigned to either remain sedentary in standard cages (Juvenile sed; n = 10/Adult sed; n = 10) or were housed in standard cages equipped with running wheels and allowed voluntary wheel access for six weeks (Juvenile run; n = 10/Adult run; n = 10). Following six weeks of wheel access, wheels were rendered immobile with metal stakes for 25 days. Daily wheel revolutions were recorded using Vital View software (Mini Mitter, Bend, OR) and running distance was calculated by multiplying the number of wheel revolutions by circumference of the wheel (1.081 m). Running distance data are represented as weekly totals. Since rats were pair-housed, values for individual rats were estimated by dividing the total weekly distance by two.

### Fecal sample collection

Fecal samples were collected from each animal at three different time points: following three days of exercise, following six weeks of exercise, and 25 days after wheels were locked. On each of the sample collection days, at approximately 0900 hours, each rat was placed into a sterile Nalgene Plexiglas cage devoid of bedding. Exposure to a novel environment has been shown to induce defecation in rats [47]. Following defecation, samples were obtained with sterilized forceps and placed into 1.5mL sterile, screw cap tubes (USA Scientific, FL), and immediately placed on ice. Forceps were sterilized with 100% ethanol between samples. Immediately following sample collection, rats were place back into their home cages and samples were frozen at -80°C until later processing.

### 16S rRNA Gene sequencing and microbial composition analysis

Samples were prepared for sequencing using established protocols [48,49]. After sample preparation, variable region 4 (V4) of 16S rRNA genes present in each sample was PCR-amplified with forward and reverse primers (F515/R806). The reverse primer is barcoded with an error-correcting 12-base Golay code to facilitate demultiplexing of up to 1,500 samples [50]. Following purification and precipitation to remove PCR artifacts, samples were subjected to multiple sequencing on an Illumina Genome Analyzer IIx. Operational taxonomic units (OTUs) were picked using a ‘closed reference’ approach [51]. In brief, this approach takes sequenced reads and compares them to a reference database. A sequence is considered a ‘hit’ if it matches something in the reference database at greater than 97% sequence identity. If an experimental sequence failed to match any member of the reference collection, it was discarded. Closed-reference picking is preferable to ‘de-novo’ or ‘open-reference’ picking in well characterized rat gut communities because the curated reference database acts as a filter; low quality or noisy sequences which get past the quality control steps, but do not actually represent novel OTUs, are eliminated. GreenGenes May 2013 version was the reference database used [52], and all sequence processing was done with QIIME v 1.8.0 [53] using the UCLUST algorithm [54]. Taxonomy and phylogeny were taken from the GreenGenes reference collection. The current experiment generated 5,787,335 sequences, of which 1,132,569 were discarded because of uncorrectable barcode errors, low quality, or for being too short (using the default parameters in the QIIME script ‘split_libraries_fastq.py’). The remaining 4,654,766 sequences of median length 151 nucleotides were clustered. The resulting OTU table was rarefied at 8468 sequences/sample to correct for uneven sequencing depth due to amplification differences between samples. Rarefaction is a conservative approach that normalizes library size to prevent type I errors in a variety of techniques applied by QIIME. Recent literature has questioned the ‘statistical admissibility’ of rarefaction [55] in the context of differential abundance testing (e.g. ANOVA), but provide a superior method for only the basic two-way comparison. PCoA, supervised learning, and other methods perform poorly without rarefaction when sequencing depth differs between samples. To check that our selected rarefaction depth was not responsible for erroneous conclusions, these data were also rarefied at higher levels to check that patterns were not artifacts of low sequence coverage. PCoA visualizations were done using the Emperor software package [56].

N = 9 samples/group were submitted for sequencing at the first two time points, and n = 6 samples/group were submitted for the final time point. Rats were excluded due insufficient fecal samples. Final group sizes used in all microbial analyses are as follows: Adult sed at three days (n = 9), six weeks (n = 7; two samples excluded due to yield less than 8468 sequences; see rarefaction description above), and 25 days post (n = 5; one sample excluded due to yield less than 8468 sequences); Adult run at three days (n = 9), six weeks (n = 9), and 25 days post (n = 6); Juvenile sed at three days (n = 9), six weeks (n = 9), and 25 days post (n = 6); Juvenile run at three days (n = 9), six weeks (n = 9), and 25 days post (n = 6).

#### Alpha diversity

Three measures of alpha diversity were calculated for all samples: Shannon entropy, an indicator of an even and balanced community structure; species richness, the observed number of species; and phylogenetic diversity, the total descending branch length of the constructed phylogenetic tree for a given sample [57].

#### Beta diversity

Principal coordinates analysis (PCoA) was performed using unweighted UniFrac distances. Briefly, UniFrac is an algorithm that determines differences between microbial communities between samples based upon their shared branch length on a phylogenetic tree [58].

#### Supervised learning

Supervised learning is a type of machine learning approach that splits data into training and test sets to build predictive models of class (sample) labels given the features (OTUs) in those samples. Here, we employ the popular random forests (RF) algorithm. In brief, the RF model utilizes a forest of decision trees to attempt to predict which experimental group a sample came from based upon the presence of certain features (OTUs and taxonomically grouped OTUs) within that sample. The supervised learning algorithm is allowed to train on a subset of samples, and is then used to classify the remainder of the samples. The success rate of this algorithm is defined by its classification accuracy, which is computed by the ratio of the percentage of mislabeled samples using random guesses / the percentage of mislabeled samples using the models of the decision trees. A classification accuracy value of 2.0 or higher indicates that particular OTUs can be used to predict what experimental group a sample came from with significantly higher accuracy than random chance, and can signify robust changes to the microbial population due to an experimental manipulation [59].

#### Data Repository

Data from the present study have been submitted to the European Bioinformatics Institute (EBI; study accession: ERP009029).

### Body composition analysis

Chemical carcass analysis was utilized to examine the long-lasting impact of exercise on body composition in a separate cohorts of rats exposed to the same protocols, wherein adult and juvenile rats were allowed voluntary access to running wheels for six weeks. Immediately following six weeks of exercise, rats were sacrificed and carcasses were frozen for later processing (4 groups: adult sed/adult run/juvenile sed/juvenile run; n = 6/group). Wheels were locked for 25 days for the remainder of the rats (4 groups; n = 6/group). 25 days following exercise cessation, these rats were also sacrificed and carcasses were frozen for later carcass analysis. Chemical carcass analysis was performed on all rats in accordance to previously published protocols [60] to determine total fat mass and total lean mass (total lean mass was calculated as fat free dry mass plus water content, minus ash content).

### Statistical Analysis

Statistical analyses were conducted using the SPSS software package V.21 (SPSS, Chicago, IL). Running distance was compared using a 2 (age) by 6 (weeks of exercise) mixed design ANOVA, and body weight was compared using a 2 (age) by 2 (exercise status) by 9 (weeks) mixed design ANOVA. Chemical carcass body composition and body weight were analyzed using a 2 (age) by 2 (exercise status) by time point (6 wks vs. 25d post). Alpha diversity measures (Shannon entropy, species richness, and phylogenic diversity) as well as relative abundance of microbial taxa at the phylum and the genus levels were subjected to normality tests (Shapiro-Wilk), and all non-normal data were subsequently rank transformed. A 2 (age) by 2 (exercise status) by 3 (time point of fecal sample collection; 3d vs. 6 wk vs. 25d post) mixed design ANOVA was then used to investigate measures of alpha diversity and relative abundance at the phylum and genus levels. At the genus level, taxa without an order classifier were excluded from analyses. Correction for multiple comparisons was conducted using the Benjamini-Hochberg step down method [61] implemented in the QIIME 1.8.0. Significant interactions were further investigated with Fisher’s PLSD, with alpha set to p<.05. Supervised machine learning using random forests as implemented in QIIME were employed to classify and differentiate sample classes.

## Results

### Running distance and body weight

**Fig 1:**
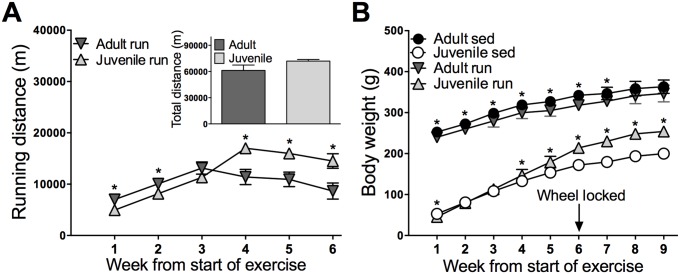
Running distance and body weight. A) Weekly total running distance across six weeks of exercise, estimated per rat. Adults ran more in the first half of exercise, whereas juveniles ran more during the second half of exercise, although total distance summed across six weeks did not differ between age groups. B) Body weight across the duration of the experiment; adult runners weighed less than their sedentary counterparts during exercise, then returned to sedentary levels shortly following exercise cessation. Juvenile runners weighed more than their sedentary counterparts toward the end of exercise and continued to weigh more after exercise cessation. Data are represented as mean ± SEM; *p<0.05.

The mean total weekly running distances, estimated per rat, were calculated for adult and juvenile runners. Running distance increased across six weeks (p<0.0001), and age did not impact total running distance (p = 0.110). A time by age interaction was observed (p<0.0001). During the initial two weeks of exercise, adult onset runners ran significantly more than juvenile onset runners; however, during the second half of the exercise, juveniles ran significantly more than adults (Fig 1A; see graph for detailed post-hoc comparisons).

Both adults and juveniles gained weight throughout the experiment (p<0.001). Overall, the adults weighed more than the juveniles (p<0.001). ANOVA also revealed significant interactions between time and age (p<0.001), time and exercise status (p<0.001), as well as age and exercise status (p<0.001). Post hoc comparisons revealed that adult onset runners weighed significantly less than their sedentary counterparts during exercise, and returned to sedentary levels one week following exercise cessation. In contrast to the pattern observed in the adults, juvenile onset runners began to weigh more during exercise, and continued to gain more weight than their sedentary counterparts following exercise cessation (Fig 1B; see graph for detailed post-hoc comparisons).

### Early life exercise and age altered alpha diversity

**Fig 2:**
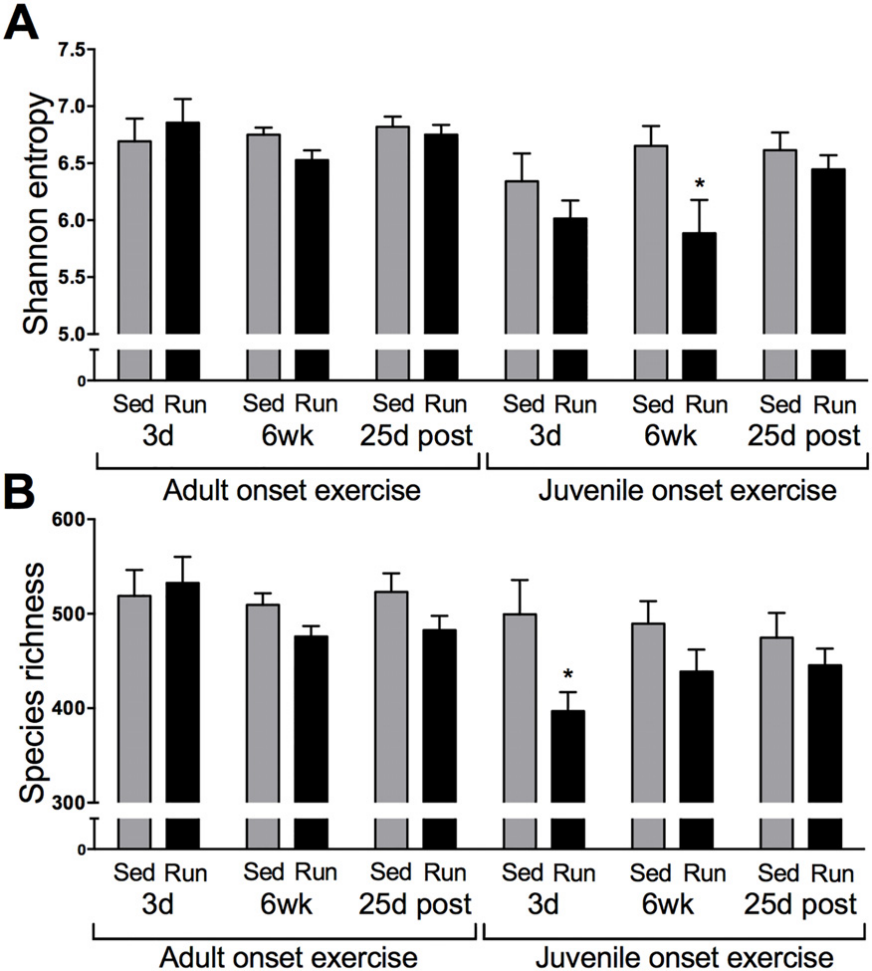
Early life exercise and age altered alpha diversity. Measures of alpha diversity for adult and juvenile run and sed rats after three days (3d) and six weeks (6 wk) of wheel running, and 25 days following exercise cessation (25d post). A) Shannon entropy, an indicator of an even community structure, was significantly higher in the adults than juveniles. Juvenile runners displayed decreased Shannon entropy overall and at 6 wk. B) Species richness was significantly higher in the adults relative to juveniles. Runners had significantly fewer species overall than their sedentary counterparts, and juvenile runners had significantly fewer species than juvenile sedentary rats 3d following the start of exercise. Data are represented as mean ± SEM; *p<0.05.

Analysis of Shannon entropy (Fig 2A) revealed that adults had higher Shannon entropy than juveniles (p<0.01), indicating that their microbial communities were more balanced and evenly dispersed. Additionally, juvenile runners displayed decreased Shannon entropy (exercise status by age interaction; p<0.05), indicating that their microbial communities were less balanced compared to their sedentary counterparts. Analyses at each time point revealed that juvenile runners showed decreased Shannon entropy after six weeks of exercise compared to sedentary juveniles. Running had no impact on Shannon entropy in adults.

Examination of species richness (Fig 2B) revealed that adults exhibited more species overall than juveniles (p<0.05), and runners had fewer species overall (p<0.05) than sedentary rats. A time by run by age interaction was also observed (p<0.05), in that juvenile runners exhibited fewer species than juvenile sedentary rats after three days of exercise. No differences were observed between adult runners and adult sedentary rats at any time point.

ANOVA revealed no statistically reliable group differences in phylogenetic diversity.

### Early life exercise and age altered beta diversity

**Fig 3:**
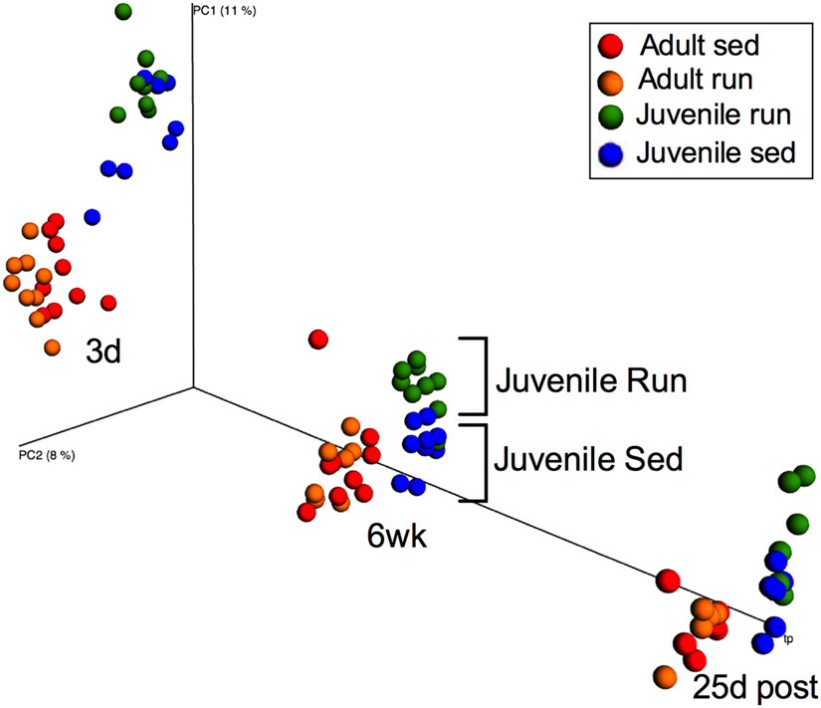
Early life exercise and age altered beta diversity. Principle coordinates analysis (PCoA) using unweighted UniFrac distances with an explicit time axis depicts clustering of microbial communities due to age after three days (3d) and six weeks (6 wk) of exercise and 25 days following exercise cessation (25d post). After 6 wk, a clear clustering of juvenile run versus juvenile sed samples is noticeable.

Principal coordinates analysis (PCoA) using unweighted UniFrac distances with an explicit time axis revealed clustering of the microbial communities of juveniles versus adults at each time point (Fig 3). After six weeks of exercise, a clear clustering of the microbial communities of juvenile runners versus juvenile sedentary rats is evident, with no noticeable pattern within the adults.

### Effects of early life exercise at the phylum level

**Fig 4:**
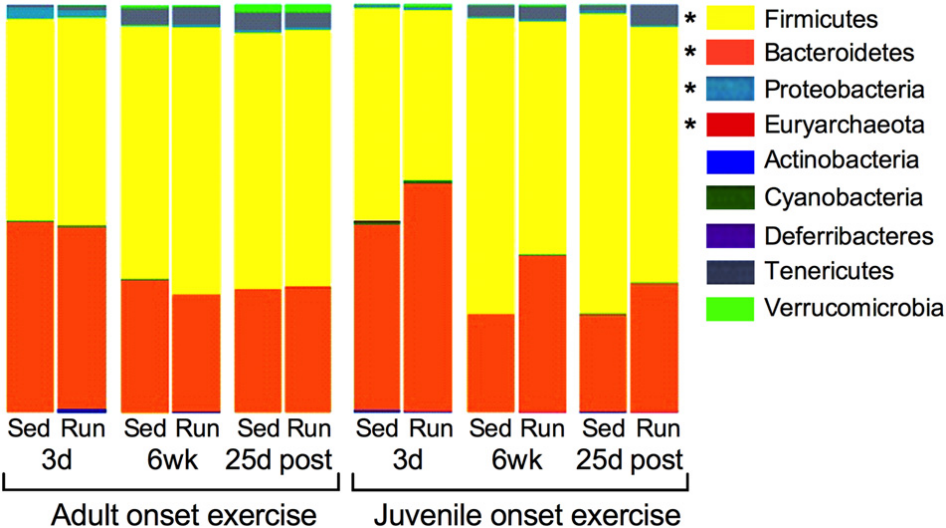
Effects of early life exercise at the phylum level. The relative abundance of nine phyla for adult and juvenile run and sed rats following three days (3d) and six weeks (6 wk) of exercise, and 25 days following exercise cessation (25d post). Significant differences in phyla due to exercise were only observed in juvenile runners. Specifically, juvenile onset exercise increased relative abundance of Euryarchaeota and Bacteroidetes and decreased relative abundance of Firmicutes and Proteobacteria, overall as well as at 6 wk. *p<0.05.

Next, the relative abundances of nine phyla were examined (Fig 4). After controlling for false discovery rate, ANOVA revealed that only Deferribacteres changed significantly across time (p = 0.0315). A time by age interaction in Actinobacteria (p<0.05) was also observed, in that higher levels were detected in the adults after six weeks. Significant differences due to exercise were only observed in juvenile runners within Firmicutes, Bacteroidetes, Proteobacteria and Euryarchaeota (exercise by age interaction; p≤0.05). Specifically, juvenile onset exercise increased relative abundance of Euryarchaeota and Bacteroidetes overall, and analyses at each time point revealed that these phyla were significantly increased compared to juvenile sedentary rats after six weeks of exercise (p<0.05). An opposite pattern was observed in Firmicutes and Proteobacteria in that these phyla both decreased in juvenile runners compared to their sedentary counterparts, overall and specifically after six weeks of exercise (p<0.05). Additionally, juvenile runners showed a trend toward a persistent decrease in Firmicutes 25 days following exercise cessation (p = 0.061).

### Supervised learning analyses: early life exercise altered specific genera

**Fig 5:**
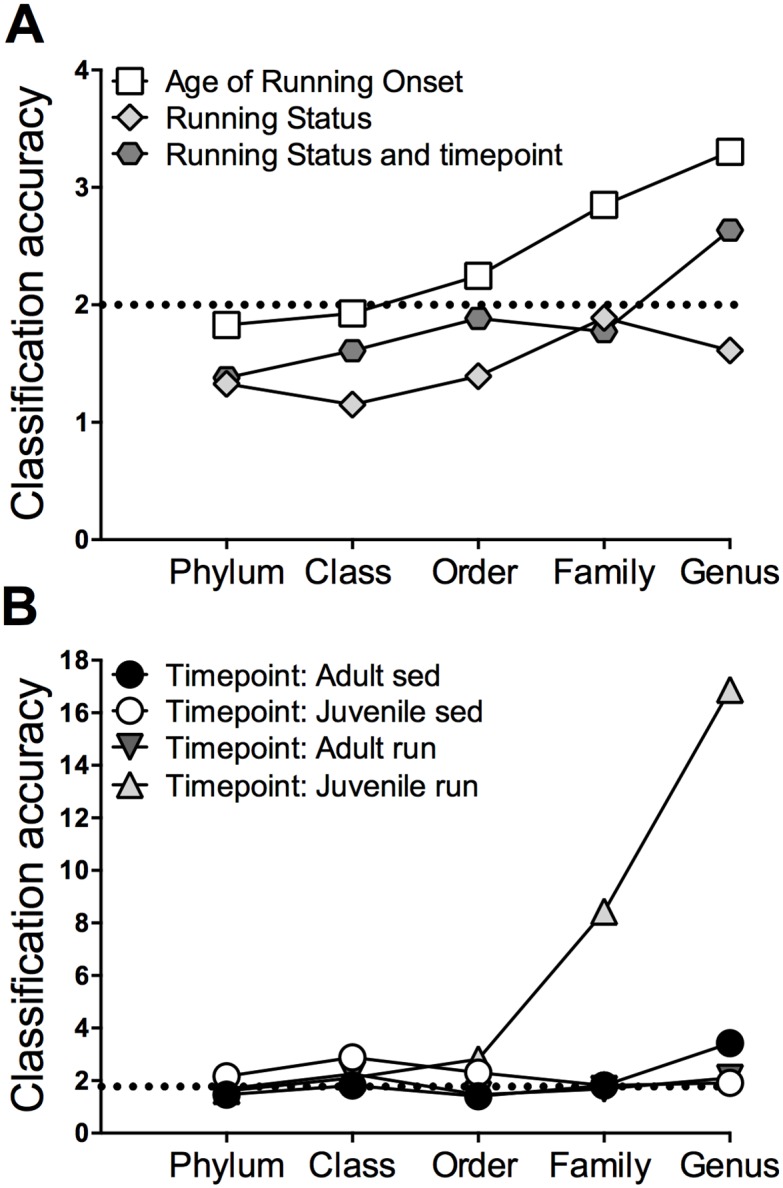
Supervised learning analyses: early life exercise altered specific genera. Classification accuracy generated from supervised learning depicted across each level of taxonomy. A) When samples were collapsed into age of running onset, running status, and running status and time point categories, the highest classification accuracy was observed for age of running onset. B) Next, the algorithm attempted to classify time of sample collection separately for each experimental group. The classification accuracy of predicting time point increased to 16.875 times better than random guessing for juvenile runners only, indicating that certain genera were significantly altered across time in juvenile runners.

Next, supervised machine learning was utilized to examine differences in microbial composition across all levels of taxonomy in order to determine if significant changes were apparent beyond the phylum level. Fig 5 depicts classification accuracy as a function of the taxonomic level of the features. When collapsing samples into the following three categories: age of running onset, running status, and running status and time point, supervised learning revealed that the highest classification accuracy was observed when samples were classified based upon age of running onset (Fig 5A), signifying pronounced differences in microbial composition between juvenile onset runners versus adult onset runners. Classification accuracy increased across levels of taxonomy and was highest for age of running onset at the genus level, indicating that a *specific* subset of genera can be used to accurately distinguish between samples belonging to juvenile versus adult runners.

Next, each experimental group was considered separately, and the supervised learning algorithm attempted to classify which time point each sample was obtained from (Fig 5B). When considering juvenile runners only, the classification accuracy of predicting time of sample collection (i.e., three days after start of exercise, six weeks after start of exercise, or 25 days post exercise) increased to 16.875 times better than random guessing at the genus level. These results indicate that a subset of specific genera served as accurate predictors of sample time point in juvenile runners only, suggesting that samples obtained from juvenile runners contained a unique microbial composition at each time point.

Supervised learning can distinguish the particular microbial taxa that are acting as the most accurate predictors of sample time point. The algorithm accomplished this by assigning an importance score to each taxon based upon the decrease in classification accuracy observed when that taxon is removed as a predictor. Here, particular taxa were considered to be highly predictive if the mean decrease in accuracy was 1.0% or more. We examined importance scores for juvenile runners at the genus level only, since prior analyses revealed that classification accuracy was highest at this level. Seven genera were identified as having an importance score of 1.0% or higher; this indicates that these genera were significantly altered by juvenile onset exercise, and served as key predictors for discerning between time points in samples obtained from juvenile runners (Table 1).

**Table 1:** Discriminant taxa and respective feature importance scores.

PHYLUM	GENUS	FEATURE IMPORTANCE SCORE
Bacteroidetes	Rikenellaceae g_	2.98%
	Parabacteroides spp.	2.97%
	Bacteroides spp.	1.40%
Firmicutes	Ruminococcus spp.	1.43%
	Christensenellaceae g_	1.22%
Actinobacteria	Bifidobacterium spp.	1.20%
Euryarchaeota	Methanosphaera spp.	1.00%

Taxa important for predicting which time point a sample obtained from a juvenile runner came from, at the genus level of taxonomy. The feature importance score of each taxon depicts the decrease in classification accuracy observed when that particular taxon is removed as a predictor. Here, a taxon was considered to be important if the mean decrease in accuracy was 1.0% or more.

### Impact of juvenile versus adult onset exercise at the genus level

Given that supervised learning analyses revealed high classification accuracy at the genus level, additional analyses at this level of taxonomy were performed. After correcting for false discovery rate, mixed design ANOVA revealed that several bacterial genera were significantly modulated by age, exercise status, and time (S1 Table). Notably, exercise by age interactions were identified in seven genera; six of these genera were significantly modulated by juvenile onset exercise, while three were modulated by adult onset exercise (Table 2).

**Table 2:** Impact of juvenile versus adult onset exercise at the genus level.

PHYLUM	GENUS	POST-FDR P VALUE	DIRECTION
Bacteroidetes	Rikenellaceae g_AF12	0.027	Decreased by juvenile exercise Increased by adult exercise
	Rikenellaceae g_	0.048	Decreased by juvenile exercise Increased by adult exercise
Firmicutes	Blautia spp.	0.02	Increased by juvenile exercise
	Turicibacter spp.	0.042	Increased by adult exercise
	Anaerostipes spp.	0.02	Increased by juvenile exercise
Euryarchaeota	Methanosphaera spp.	0.042	Increased by juvenile exercise
Proteobacteria	Desulfovibrio spp.	0.042	Decreased by juvenile exercise

Summary of exercise by age interactions observed following ANOVA at the genus level of taxonomy.

### Effects of age of exercise onset on body composition across the lifespan

**Fig 6:**
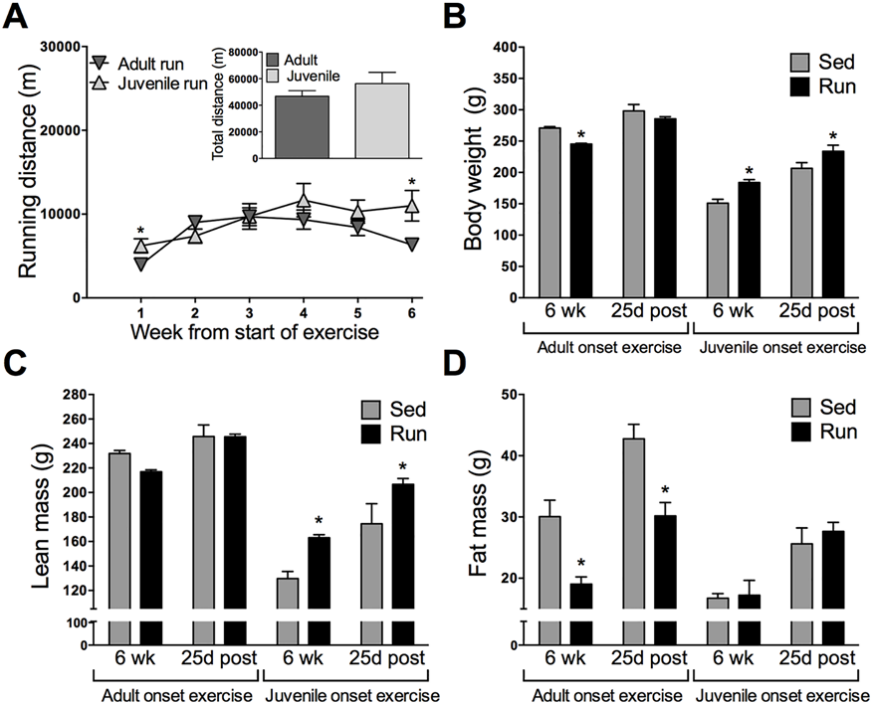
Effects of age of exercise onset on body composition across the lifespan. A) Weekly total running distance across time for the separate cohort used for chemical carcass analyses. Adult runners ran less toward the end of exercise, whereas running distance steadily increased for juvenile runners throughout the duration of exercise. Total distance summed across six weeks did not differ by age. B-D depict body weight, lean mass and fat mass, respectively, for juvenile and adult run and sed chemical carcass rats after six weeks of exercise (6 wk) or 25 days following exercise cessation (25d post). B) Body weight; adult runners weighed less than adult seds at 6 wk, however they returned to sedentary levels 25d post. In contrast, juvenile onset runners consistently weighed more than their sedentary counterparts at both time points. C) Lean mass; juvenile onset runners had sustained increases in lean mass at both time points. D) Fat mass; sustained decreases in fat mass were observed in adult onset runners only. Data are represented as mean ± SEM; *p<0.05.

Given the potential for early life exercise-induced microbiota changes to contribute to a lean phenotype, we examined body composition in juvenile versus adult run and sedentary rats using chemical carcass analysis in a separate cohort subjected to the same protocols. Mean total weekly running distances for chemical carcass rats were examined across six weeks of exercise (Fig 6A). Running distance increased overall across six weeks (p<0.0001), and age did not impact total running distance (p = 0.325). A time by age interaction was observed (p<0.0001). Similar to the running pattern observed in the 16S cohort, running distance for adult runners increased across time during the first half of exercise and declined during the second half of exercise. Conversely, running distance for juvenile runners generally increased each week throughout the duration of exercise (see graph for detailed post-hoc comparisons).

Body weights were examined immediately before chemical carcass procedures in sacrificed adult and juvenile runners after six weeks of exercise or 25 days following exercise cessation (Fig 6B). Overall, adults weighed more than juveniles (p<0.001). ANOVA also revealed significant interactions between age and exercise status (p<0.001). Post hoc comparisons revealed that adult onset runners weighed significantly less than their sedentary counterparts immediately after exercise cessation (p< 0.01) and returned to sedentary levels 25 days following exercise cessation. In contrast to the pattern observed in the adults, juvenile runners weighed more than their sedentary counterparts immediately after exercise cessation (p< 0.002), as well as 25 days following exercise cessation (p<0.007).

Carcass analysis revealed that lean mass increased across time (p<0.0001), and was higher overall in adults (p<0.0001) and runners (p<0.0108). ANOVA further revealed an age by exercise interaction (p<0.0001). Follow up analyses showed that lean mass significantly increased in juvenile runners only, following six weeks of exercise (p<0.0003) and 25 days following exercise cessation (p<0.0001), indicating that early life exercise can produce lasting increases in lean mass. No such patterns were observed in the adult runners (Fig 6C). Carcass analysis also revealed that fat mass increased across time (p<0.0001), and was higher in adults (p<0.0001), and sedentary rats (p<0.0002). ANOVA further revealed a run by age interaction (p<0.0005), in that fat mass was greater in adult sedentary rats at both time points (p<0.0012, p<0.0001, respectively), indicating that the impact of being sedentary on fat mass was greater for adults than juveniles (Fig 6D).

## Discussion

Findings from measures of alpha and beta diversity, supervised learning, and microbial composition analyses at the phylum and genus levels collectively support the hypothesis that exercise initiated during the juvenile period had a more robust impact on the gut microbiota than exercise initiated in adulthood. This point is best illustrated by comparisons at the phylum level, where changes in phyla were only observed in the juvenile runners, and with comparison at the genus level, where supervised learning and ANOVA analyses both demonstrated that microbial genera were more robustly altered in juvenile runners than in adult runners. Although several studies have demonstrated that exercise is capable of altering the gut flora [33,35,37,62–65], this study is the first to demonstrate that the gut microbiota may be more sensitive to exercise during early life.

At the phylum level, early life exercise increased the relative abundance of Bacteroidetes and decreased Firmicutes. Increased Bacteroidetes along with decreased Firmicutes within the gut may be reflective of a lean phenotype, and has been associated with adaptive metabolic consequences such as increased SCFA production, increased energy expenditure, and inhibited fat accumulation in adipose tissue [10]. Conversely, increases in Firmicutes and decreases in Bacteroidetes have been associated with obesity [12]. Interestingly, in a recent study [37], exercise prevented high-fat diet induced weight gain and similarly produced an increased Bacteroidetes to Firmicutes ratio. Importantly, in the current study, the microbial pattern reflective of a lean phenotype was only observed in the juvenile runners, indicating that the developmental stage during which exercise is initiated may be important for establishing this adaptive change in phyla. Additionally, there is tentative evidence for lasting decreases in Firmicutes in juvenile runners only (p = 0.061; 25 days following exercise cessation), suggesting that the exercise-induced changes observed within juvenile runners may be capable of persisting. This trend toward persistent changes following early life exercise warrants further investigation.

Supervised learning analyses revealed that exercise uniquely altered specific genera within the juvenile runners. Specifically, supervised learning analyses using the random forests (RF) algorithm examined differences in microbial composition between treatment groups at all levels of taxonomy. Our approach suggested that a specific subset of genera could be used to accurately classify samples belonging to juvenile versus adult runners, indicating differences in microbial composition between these two groups. Furthermore, this analysis showed that specific genera could be used to identify time of sample collection in young runners with significantly greater accuracy than any other experimental group. This suggests that the microbial communities of juvenile runners were distinctively altered at each time point, while the microbial communities of the other groups were less affected.

At the genus level, supervised learning feature importance scores identified seven discriminant genera in the juvenile runners, and ANOVA identified six genera altered by juvenile exercise and three altered by adult exercise. Among the genera identified with supervised learning, *Bifidobacteria spp*. have been linked to reducing anxiety [66] and depression [67], and have been associated with leanness in humans [34]. Notably, increases in *Bifidobacteria spp*. as well as increases in lean mass were observed in rats fed diets containing whey protein isolate [68]. *Methanosphaera spp*. were shown to be altered in young runners using both analyses; these species belong to the domain Archea and utilize hydrogen as an energy source [69]. Increases in hydrogen within the gut can hinder the efficiency of microbial fermentation, and *Methanosphaera spp*. can help provide more efficient carbohydrate fermentation. Thus, comparisons at the genus level also suggest that early life exercise can modulate specific bacteria capable of producing adaptive changes in metabolism.

Others have also observed alterations in various genera following exercise in adults [63] and the present study is the first to demonstrate that more genera are impacted by juvenile onset exercise compared to adult onset exercise. Though no changes at the phylum level were found in adults, exercise-induced changes at this level in adult humans and rats have been previously reported [62,63]. Differences in age of running onset as well as duration and type of exercise may account for this. The present data suggest that although six weeks of adult onset exercise can alter the abundance of a few genera, early onset exercise is more capable of impacting the overall structure of the microbial ecosystem.

These phylum and genus level changes are consistent with the types of phenotypic changes in body composition found in our juvenile runners. Juvenile onset runners but not adult runners showed increases in lean body mass measured using chemical carcass analyses that persisted after running had stopped, consistent with previous reports [70]. Though these effects could be attributed to exercise directly, these data collectively suggest that microbial composition in younger rats may play a role in promoting and/or maintaining sustained increases in lean mass. In support of this idea, a recent study showed that brief antibiotic regimens during early life that transiently disrupted the gut microbiota were capable of producing lasting alterations in body composition [24]. Similarly, previous work in humans has also detected a relationship between early life microbial composition and body mass later in life [25], as well as between early life microbial disruptions and obesity [26,27,71]. Although mechanisms for how exercise-altered microbial composition can promote stable changes in lean mass were not investigated in this paper, several positive metabolic consequences associated with the microbiota could play a role. For instance, an exercise-altered microbial composition could promote SCFA production and thus enhance energy availability and expenditure, as well as reduce fat storage through a variety of mechanisms [72], including modulating expression of angiopoietin-like protein 4 or ANGPTL4 [73] and decreasing lipoprotein lipase mediated triglyceride uptake [74]. Given that a number of previous studies have found a strong association between early life microbial composition and body mass throughout the lifespan, the role of the microbiota in promoting early life-exercise induced increases in lean body mass should be further explored.

Measures of alpha diversity revealed that juvenile rats had lower species richness (fewer species) as well as lower Shannon entropy (less evenly dispersed microbial communities) relative to adults. Similar patterns were found in humans across different ages [30], in that the microbial composition of infants and children was also less stable and diverse relative to adults. The increased stability and complexity of the adult microbiota may make it more impervious to environmentally-induced change [28], while the decreased stability and diversity of the younger gut may be why the early microbial environment is *more* sensitive to change. Indeed, a recent paper demonstrated that an individual’s bacterial diversity was indicative of its responsiveness to diet-induced changes; greater diversity was associated with a less responsive gut microbiota [75]. These data offer additional support for this idea.

Measures of alpha diversity further revealed that community evenness and richness were both decreased in juvenile runners. These reductions support our hypothesis that juvenile onset exercise had a greater impact on gut microbial composition than adult onset exercise. A lower Shannon value indicates an uneven community structure, suggesting that the relative abundance of some taxa is either much higher or much lower compared to other taxa. Furthermore, a complimentary lower richness value may suggest that exercise is affecting community evenness by reducing or eliminating specific taxa. These findings differ from existing findings examining richness following exercise. Clarke *et al*. reported an increase in several measures of alpha diversity in young adult professional rugby players [62]. In rats, Petriz *et al*., investigated the impact of four weeks of exercise in several rat strains and found that bacterial diversity increased after exercise across all strains [63]. Factors such as age, diet, exercise intensity and duration as well as rat strain/phenotype may account for this difference. Additionally, it is important to note that although increased diversity and richness has been linked to better health [76], these data need to be interpreted in the context of our other findings. Analyses at the phylum and genus levels reveal that while early onset exercise decreased overall species richness, it increased certain adaptive microbial phyla and genera. Thus, the patterns of change as well as the potential functional consequences are important to consider.

One of the limitations of this study is that microbial composition was only measured in fecal samples. Although we have previously found no differences between cecal contents and fecal samples using 16S [49], it is possible that samples of adherent microbes from the intestinal lumen could have yielded different results [77]. Another potential limitation of this work is that caloric intake was not measured. Because rats can regulate energy balance and typically consume the appropriate amount of calories to sustain optimal body mass [78,79], it is not surprising that rats with wheel access may ingest slightly more calories [37]. Nonetheless, although diet is an important modulator of microbiota, some studies suggest that minor, transient differences in caloric intake, in absence of manipulating the composition or nutrient quality of diet, have little impact on microbial composition [80]. Finally, possible physiological consequences of altered microbial composition were not directly investigated. While body composition was explored, body mass and bacterial composition were measured in separate cohorts of rats, making causal conclusions concerning the functional consequences of gut microbial community structure not possible. In addition, SCFA production was not investigated and should be explored in future studies.

The present study demonstrated the novel finding that exercise initiated during early life may have a more pronounced impact on the gut microbiota than exercise initiated in adulthood. These results offer support for the idea that the microbiota is more plastic and sensitive to change during early life. In addition, these results add to the growing literature demonstrating that manipulating microbial ecology in early life may produce lasting changes in host physiology [21,24,26]. Given that juvenile onset exercise produced microbial patterns associated with leanness as well as enduring increases in lean mass, it is possible that exercise-induced alterations in microbiota during early life may potentially contribute to sustained metabolic consequences.

## Supporting Information

S1 Table ANOVA main effects summary at the genus level. Summary of main effects observed following ANOVA at the genus level of taxonomy. These genera were differentially impacted by time, age, and exercise.
(DOCX)

## References

[1] Eckburg PB, Bik EM, Bernstein CN, Purdom E, Dethlefsen L, Sargent M, et al Diversity of the human intestinal microbial flora. Science. 2005;308: 1635–1638. 15831718 10.1126/science.1110591 PMC1395357

[2] Hill MJ. Intestinal flora and endogenous vitamin synthesis. Eur J Cancer Prev. 1997;6 Suppl 1: S43–45. 9167138 10.1097/00008469-199703001-00009

[3] Conly JM, Stein K, Worobetz L, Rutledge-Harding S. The contribution of vitamin K2 (menaquinones) produced by the intestinal microflora to human nutritional requirements for vitamin K. Am J Gastroenterol. 1994;89: 915–923. 8198105

[4] Sommer F, Backhed F. The gut microbiota—masters of host development and physiology. Nat Rev Microbiol. 2013;11: 227–238. 10.1038/nrmicro2974 23435359

[5] Hrncir T, Stepankova R, Kozakova H, Hudcovic T, Tlaskalova-Hogenova H. Gut microbiota and lipopolysaccharide content of the diet influence development of regulatory T cells: studies in germ-free mice. BMC Immunol. 2008;9: 65 10.1186/1471-2172-9-65 18990206 PMC2588440

[6] Tlaskalova-Hogenova H, Stepankova R, Hudcovic T, Tuckova L, Cukrowska B, Lodinova-Zadnikova R, et al Commensal bacteria (normal microflora), mucosal immunity and chronic inflammatory and autoimmune diseases. Immunol Lett. 2004;93: 97–108. 15158604 10.1016/j.imlet.2004.02.005

[7] Ohara Y, McCarron RM, Hoffman TT, Sugano H, Bembry J, Lenz FA, et al Adrenergic mediation of TNF alpha-stimulated ICAM-1 expression on human brain microvascular endothelial cells. Acta Neurochir Suppl. 2000;76: 117–120. 11449988 10.1007/978-3-7091-6346-7_24

[8] Hooper LV, Stappenbeck TS, Hong CV, Gordon JI. Angiogenins: a new class of microbicidal proteins involved in innate immunity. Nat Immunol. 2003;4: 269–273. 12548285 10.1038/ni888

[9] Berg RD, Garlington AW. Translocation of certain indigenous bacteria from the gastrointestinal tract to the mesenteric lymph nodes and other organs in a gnotobiotic mouse model. Infect Immun. 1979;23: 403–411. 154474 10.1128/iai.23.2.403-411.1979 PMC414179

[10] Ridaura VK, Faith JJ, Rey FE, Cheng J, Duncan AE, Kau AL, et al Gut microbiota from twins discordant for obesity modulate metabolism in mice. Science. 2013;341: 1241214 10.1126/science.1241214 24009397 PMC3829625

[11] Backhed F, Ding H, Wang T, Hooper LV, Koh GY, Nagy A, et al The gut microbiota as an environmental factor that regulates fat storage. Proc Natl Acad Sci U S A. 2004;101: 15718–15723. 15505215 10.1073/pnas.0407076101 PMC524219

[12] Turnbaugh PJ, Hamady M, Yatsunenko T, Cantarel BL, Duncan A, Ley RE, et al A core gut microbiome in obese and lean twins. Nature. 2009;457: 480–484. 10.1038/nature07540 19043404 PMC2677729

[13] Turnbaugh PJ, Ley RE, Mahowald MA, Magrini V, Mardis ER, Gordon JI. An obesity-associated gut microbiome with increased capacity for energy harvest. Nature. 2006;444: 1027–1031. 17183312 10.1038/nature05414

[14] Duncan SH, Lobley GE, Holtrop G, Ince J, Johnstone AM, Louis P, et al Human colonic microbiota associated with diet, obesity and weight loss. Int J Obes (Lond). 2008;32: 1720–1724. 10.1038/ijo.2008.155 18779823

[15] Schwiertz A, Taras D, Schafer K, Beijer S, Bos NA, Donus C, et al Microbiota and SCFA in lean and overweight healthy subjects. Obesity (Silver Spring). 2010;18: 190–195. 10.1038/oby.2009.167 19498350

[16] Round JL, Mazmanian SK. Inducible Foxp3+ regulatory T-cell development by a commensal bacterium of the intestinal microbiota. Proc Natl Acad Sci U S A. 2010;107: 12204–12209. 10.1073/pnas.0909122107 20566854 PMC2901479

[17] Atarashi K, Tanoue T, Shima T, Imaoka A, Kuwahara T, Momose Y, et al Induction of colonic regulatory T cells by indigenous Clostridium species. Science. 2011;331: 337–341. 10.1126/science.1198469 21205640 PMC3969237

[18] Bravo JA, Forsythe P, Chew MV, Escaravage E, Savignac HM, Dinan TG, et al Ingestion of Lactobacillus strain regulates emotional behavior and central GABA receptor expression in a mouse via the vagus nerve. Proc Natl Acad Sci U S A. 2011;108: 16050–16055. 10.1073/pnas.1102999108 21876150 PMC3179073

[19] Messaoudi M, Lalonde R, Violle N, Javelot H, Desor D, Nejdi A, et al Assessment of psychotropic-like properties of a probiotic formulation (Lactobacillus helveticus R0052 and Bifidobacterium longum R0175) in rats and human subjects. Br J Nutr. 2011;105: 755–764. 10.1017/S0007114510004319 20974015

[20] Rao AV, Bested AC, Beaulne TM, Katzman MA, Iorio C, Berardi JM, et al A randomized, double-blind, placebo-controlled pilot study of a probiotic in emotional symptoms of chronic fatigue syndrome. Gut Pathog. 2009;1: 6 10.1186/1757-4749-1-6 19338686 PMC2664325

[21] Sudo N, Chida Y, Aiba Y, Sonoda J, Oyama N, Yu XN, et al Postnatal microbial colonization programs the hypothalamic-pituitary-adrenal system for stress response in mice. J Physiol. 2004;558: 263–275. 15133062 10.1113/jphysiol.2004.063388 PMC1664925

[22] Diaz Heijtz R, Wang S, Anuar F, Qian Y, Bjorkholm B, Samuelsson A, et al Normal gut microbiota modulates brain development and behavior. Proc Natl Acad Sci U S A. 2011;108: 3047–3052. 10.1073/pnas.1010529108 21282636 PMC3041077

[23] Sudo N, Sawamura S, Tanaka K, Aiba Y, Kubo C, Koga Y. The requirement of intestinal bacterial flora for the development of an IgE production system fully susceptible to oral tolerance induction. J Immunol. 1997;159: 1739–1745. 9257835

[24] Cox LM, Yamanishi S, Sohn J, Alekseyenko AV, Leung JM, Cho I, et al Altering the intestinal microbiota during a critical developmental window has lasting metabolic consequences. Cell. 2014;158: 705–721. 10.1016/j.cell.2014.05.052 25126780 PMC4134513

[25] Kalliomaki M, Collado MC, Salminen S, Isolauri E. Early differences in fecal microbiota composition in children may predict overweight. Am J Clin Nutr. 2008;87: 534–538. 18326589 10.1093/ajcn/87.3.534

[26] Ajslev TA, Andersen CS, Gamborg M, Sorensen TI, Jess T. Childhood overweight after establishment of the gut microbiota: the role of delivery mode, pre-pregnancy weight and early administration of antibiotics. Int J Obes (Lond). 2011;35: 522–529. 10.1038/ijo.2011.27 21386800

[27] Murphy R, Stewart AW, Braithwaite I, Beasley R, Hancox RJ, Mitchell EA, et al Antibiotic treatment during infancy and increased body mass index in boys: an international cross-sectional study. Int J Obes (Lond). 2014;38: 1115–1119. 10.1038/ijo.2013.218 24257411

[28] Wall R, Ross RP, Ryan CA, Hussey S, Murphy B, Fitzgerald GF, et al Role of gut microbiota in early infant development. Clin Med Pediatr. 2009;3: 45–54. 23818794 10.4137/cmped.s2008 PMC3676293

[29] O'Toole PW, Claesson MJ. Gut microbiota: Changes throughout the lifespan from infancy to elderly International Dairy Journal 2010;: 281–291.

[30] Yatsunenko T, Rey FE, Manary MJ, Trehan I, Dominguez-Bello MG, Contreras M, et al Human gut microbiome viewed across age and geography. Nature. 2012;486: 222–227. 10.1038/nature11053 22699611 PMC3376388

[31] Palmer C, Bik EM, DiGiulio DB, Relman DA, Brown PO. Development of the human infant intestinal microbiota. PLoS Biol. 2007;5: e177 17594176 10.1371/journal.pbio.0050177 PMC1896187

[32] Koenig JE, Spor A, Scalfone N, Fricker AD, Stombaugh J, Knight R, et al Succession of microbial consortia in the developing infant gut microbiome. Proc Natl Acad Sci U S A. 2011;108 Suppl 1: 4578–4585. 10.1073/pnas.1000081107 20668239 PMC3063592

[33] Queipo-Ortuno MI, Seoane LM, Murri M, Pardo M, Gomez-Zumaquero JM, Cardona F, et al Gut microbiota composition in male rat models under different nutritional status and physical activity and its association with serum leptin and ghrelin levels. PLoS One. 2013;8: e65465 10.1371/journal.pone.0065465 23724144 PMC3665787

[34] Million M, Angelakis E, Maraninchi M, Henry M, Giorgi R, Valero R, et al Correlation between body mass index and gut concentrations of Lactobacillus reuteri, Bifidobacterium animalis, Methanobrevibacter smithii and Escherichia coli. International Journal of Obesity. 2013;37: 1460–1466. 10.1038/ijo.2013.20 23459324 PMC3826031

[35] Matsumoto M, Inoue R, Tsukahara T, Ushida K, Chiji H, Matsubara N, et al Voluntary running exercise alters microbiota composition and increases n-butyrate concentration in the rat cecum. Biosci Biotechnol Biochem. 2008;72: 572–576. 18256465 10.1271/bbb.70474

[36] Gao Z, Yin J, Zhang J, Ward RE, Martin RJ, Lefevre M, et al Butyrate improves insulin sensitivity and increases energy expenditure in mice. Diabetes. 2009;58: 1509–1517. 10.2337/db08-1637 19366864 PMC2699871

[37] Evans CC, LePard KJ, Kwak JW, Stancukas MC, Laskowski S, Dougherty J, et al Exercise prevents weight gain and alters the gut microbiota in a mouse model of high fat diet-induced obesity. PLoS One. 2014;9: e92193 10.1371/journal.pone.0092193 24670791 PMC3966766

[38] Greenwood BN, Foley TE, Le TV, Strong PV, Loughridge AB, Day HE, et al Long-term voluntary wheel running is rewarding and produces plasticity in the mesolimbic reward pathway. Behav Brain Res. 2011;217: 354–362. 10.1016/j.bbr.2010.11.005 21070820 PMC3021978

[39] Mann PB, Jiang W, Zhu Z, Wolfe P, McTiernan A, Thompson HJ. Wheel running, skeletal muscle aerobic capacity and 1-methyl-1-nitrosourea induced mammary carcinogenesis in the rat. Carcinogenesis. 2010;31: 1279–1283. 10.1093/carcin/bgq063 20299525 PMC2893798

[40] Speaker KJ, Cox SS, Paton MM, Serebrakian A, Maslanik T, Greenwood BN, et al Six weeks of voluntary wheel running modulates inflammatory protein (MCP-1, IL-6, and IL-10) and DAMP (Hsp72) responses to acute stress in white adipose tissue of lean rats. Brain Behav Immun. 2014;39: 87–98. 10.1016/j.bbi.2013.10.028 24246250 PMC4301739

[41] Greenwood BN, Fleshner M. Exercise, learned helplessness, and the stress-resistant brain. Neuromolecular Med. 2008;10: 81–98. 10.1007/s12017-008-8029-y 18300002

[42] Greenwood BN, Foley TE, Burhans D, Maier SF, Fleshner M. The consequences of uncontrollable stress are sensitive to duration of prior wheel running. Brain Res. 2005;1033: 164–178. 15694921 10.1016/j.brainres.2004.11.037

[43] Greenwood BN, Foley TE, Day HE, Campisi J, Hammack SH, Campeau S, et al Freewheel running prevents learned helplessness/behavioral depression: role of dorsal raphe serotonergic neurons. J Neurosci. 2003;23: 2889–2898. 12684476 10.1523/JNEUROSCI.23-07-02889.2003 PMC6742115

[44] Greenwood BN, Loughridge AB, Sadaoui N, Christianson JP, Fleshner M. The protective effects of voluntary exercise against the behavioral consequences of uncontrollable stress persist despite an increase in anxiety following forced cessation of exercise. Behav Brain Res. 2012;233: 314–321. 10.1016/j.bbr.2012.05.017 22610051 PMC3402647

[45] Meijer JH, Robbers Y. Wheel running in the wild. Proc Biol Sci. 2014;281. 10.1098/rspb.2014.0210 PMC4046404 24850923

[46] Takemoto TI, Suzuki T, Miyama T. Effects of isolation on mice in relation to age and sex. Tohoku J Exp Med. 1975;117: 153–165. 1209605 10.1620/tjem.117.153

[47] Restrepo C, Armario A. Chronic stress alters pituitary-adrenal function in prepubertal male rats. Psychoneuroendocrinology. 1987;12: 393–398. 2829263 10.1016/0306-4530(87)90068-0

[48] Caporaso JG, Lauber CL, Costello EK, Berg-Lyons D, Gonzalez A, Stombaugh J, et al Moving pictures of the human microbiome. Genome Biol. 2011;12: R50 10.1186/gb-2011-12-5-r50 21624126 PMC3271711

[49] Maslanik T, Tannura K, Mahaffey L, Loughridge AB, Benninson L, Ursell L, et al Commensal bacteria and MAMPs are necessary for stress-induced increases in IL-1beta and IL-18 but not IL-6, IL-10 or MCP-1. PLoS One. 2012;7: e50636 10.1371/journal.pone.0050636 23236381 PMC3517493

[50] Caporaso JG, Lauber CL, Walters WA, Berg-Lyons D, Lozupone CA, Turnbaugh PJ, et al Global patterns of 16S rRNA diversity at a depth of millions of sequences per sample. Proc Natl Acad Sci U S A. 2011;108 Suppl 1: 4516–4522. 10.1073/pnas.1000080107 20534432 PMC3063599

[51] Navas-Molina JA, Peralta-Sanchez JM, Gonzalez A, McMurdie PJ, Vazquez-Baeza Y, Xu Z, et al Advancing our understanding of the human microbiome using QIIME. Methods Enzymol. 2013;531: 371–444. 10.1016/B978-0-12-407863-5.00019-8 24060131 PMC4517945

[52] McDonald D, Price MN, Goodrich J, Nawrocki EP, DeSantis TZ, Probst A, et al An improved Greengenes taxonomy with explicit ranks for ecological and evolutionary analyses of bacteria and archaea. ISME J. 2012;6: 610–618. 10.1038/ismej.2011.139 22134646 PMC3280142

[53] Caporaso JG, Kuczynski J, Stombaugh J, Bittinger K, Bushman FD, Costello EK, et al QIIME allows analysis of high-throughput community sequencing data. Nat Methods. 2010;7: 335–336. 10.1038/nmeth.f.303 20383131 PMC3156573

[54] Edgar RC. Search and clustering orders of magnitude faster than BLAST. Bioinformatics. 2010;26: 2460–2461. 10.1093/bioinformatics/btq461 20709691

[55] McMurdie PJ, Holmes S. Waste not, want not: why rarefying microbiome data is inadmissible. PLoS Comput Biol. 2014;10: e1003531 10.1371/journal.pcbi.1003531 24699258 PMC3974642

[56] Vazquez-Baeza Y, Pirrung M, Gonzalez A, Knight R. EMPeror: a tool for visualizing high-throughput microbial community data. Gigascience. 2013;2: 16 10.1186/2047-217X-2-16 24280061 PMC4076506

[57] Faith DP. Conservation evaluation and phylogenic diversity. Biological Conservation. 1992;61: 1–10.

[58] Lozupone C, Knight R. UniFrac: a new phylogenetic method for comparing microbial communities. Appl Environ Microbiol. 2005;71: 8228–8235. 16332807 10.1128/AEM.71.12.8228-8235.2005 PMC1317376

[59] Knights D, Costello EK, Knight R. Supervised classification of human microbiota. FEMS Microbiol Rev. 2011;35: 343–359. 10.1111/j.1574-6976.2010.00251.x 21039646

[60] Smith D Jr., Johnson M, Nagy T. Precision and accuracy of bioimpedance spectroscopy for determination of in vivo body composition in rats. Int J Body Compos Res. 2009;7: 21–26. 19668348 PMC2722071

[61] Benjamini Y, Hochberg Y. Controlling the false discovery rate: A practical and powerful approach to multiple testing. Journal of the Royal Statistical Society Series B (Methodological). 1995;57: 289–300.

[62] Clarke SF, Murphy EF, O'Sullivan O, Lucey AJ, Humphreys M, Hogan A, et al Exercise and associated dietary extremes impact on gut microbial diversity. Gut. 2014. 10.1136/gutjnl-2013-306541 25021423

[63] Petriz BA, Castro AP, Almeida JA, Gomes CP, Fernandes GR, Kruger RH, et al Exercise induction of gut microbiota modifications in obese, non-obese and hypertensive rats. BMC Genomics. 2014;15: 511 10.1186/1471-2164-15-511 24952588 PMC4082611

[64] Kang SS, Jeraldo PR, Kurti A, Miller ME, Cook MD, Whitlock K, et al Diet and exercise orthogonally alter the gut microbiome and reveal independent associations with anxiety and cognition. Mol Neurodegener. 2014;9: 36 10.1186/1750-1326-9-36 25217888 PMC4168696

[65] Santacruz A, Marcos A, Warnberg J, Marti A, Martin-Matillas M, Campoy C, et al Interplay between weight loss and gut microbiota composition in overweight adolescents. Obesity (Silver Spring). 2009;17: 1906–1915. 10.1038/oby.2009.112 19390523

[66] Bercik P, Park AJ, Sinclair D, Khoshdel A, Lu J, Huang X, et al The anxiolytic effect of Bifidobacterium longum NCC3001 involves vagal pathways for gut-brain communication. Neurogastroenterol Motil. 2011;23: 1132–1139. 10.1111/j.1365-2982.2011.01796.x 21988661 PMC3413724

[67] Desbonnet L, Garrett L, Clarke G, Bienenstock J, Dinan TG. The probiotic Bifidobacteria infantis: An assessment of potential antidepressant properties in the rat. J Psychiatr Res. 2008;43: 164–174. 10.1016/j.jpsychires.2008.03.009 18456279

[68] McAllan L, Skuse P, Cotter PD, O'Connor P, Cryan JF, Ross RP, et al Protein quality and the protein to carbohydrate ratio within a high fat diet influences energy balance and the gut microbiota in C57BL/6J mice. PLoS One. 2014;9: e88904 10.1371/journal.pone.0088904 24520424 PMC3919831

[69] Wilson M Microbial inhabitants of humans: Their ecology and role in health and disease. Cambridge, UK: Cambridge University Press; 2005.

[70] Shindo D, Matsuura T, Suzuki M. Effects of prepubertal-onset exercise on body weight changes up to middle age in rats. J Appl Physiol (1985). 2014;116: 674–682. 10.1152/japplphysiol.00405.2013 24458753

[71] Trasande L, Blustein J, Liu M, Corwin E, Cox LM, Blaser MJ. Infant antibiotic exposures and early-life body mass. Int J Obes (Lond). 2013;37: 16–23. 10.1038/ijo.2012.132 22907693 PMC3798029

[72] Tremaroli V, Backhed F. Functional interactions between the gut microbiota and host metabolism. Nature. 2012;489: 242–249. 10.1038/nature11552 22972297

[73] Korecka A, de Wouters T, Cultrone A, Lapaque N, Pettersson S, Dore J, et al ANGPTL4 expression induced by butyrate and rosiglitazone in human intestinal epithelial cells utilizes independent pathways. Am J Physiol Gastrointest Liver Physiol. 2013;304: G1025–1037. 10.1152/ajpgi.00293.2012 23518684

[74] Aronsson L, Huang Y, Parini P, Korach-Andre M, Hakansson J, Gustafsson JA, et al Decreased fat storage by Lactobacillus paracasei is associated with increased levels of angiopoietin-like 4 protein (ANGPTL4). PLoS One. 2010;5. 10.1371/journal.pone.0013087 PMC2948012 20927337

[75] Salonen A, Lahti L, Salojarvi J, Holtrop G, Korpela K, Duncan SH, et al Impact of diet and individual variation on intestinal microbiota composition and fermentation products in obese men. ISME J. 2014. 10.1038/ismej.2014.63 PMC4992075 24763370

[76] Bisgaard H, Li N, Bonnelykke K, Chawes BL, Skov T, Paludan-Muller G, et al Reduced diversity of the intestinal microbiota during infancy is associated with increased risk of allergic disease at school age. J Allergy Clin Immunol. 2011;128: 646–652 e641–645. 10.1016/j.jaci.2011.04.060 21782228

[77] Haange SB, Oberbach A, Schlichting N, Hugenholtz F, Smidt H, von Bergen M, et al Metaproteome analysis and molecular genetics of rat intestinal microbiota reveals section and localization resolved species distribution and enzymatic functionalities. J Proteome Res. 2012;11: 5406–5417. 10.1021/pr3006364 23016992

[78] Stevenson JA, Box BM, Feleki V, Beaton JR. Bouts of exercise and food intake in the rat. J Appl Physiol. 1966;21: 118–122. 5903897 10.1152/jappl.1966.21.1.118

[79] Adolph EF. Urges to eat and drink in rats. Am J Physiol. 1947;151: 110–125. 20271650 10.1152/ajplegacy.1947.151.1.110

[80] Mai V, Colbert LH, Perkins SN, Schatzkin A, Hursting SD. Intestinal microbiota: a potential diet-responsive prevention target in ApcMin mice. Mol Carcinog. 2007;46: 42–48. 16929480 10.1002/mc.20233

