# Supplementary material for: Exercise Is More Effective at Altering Gut Microbial Composition and Producing Stable Changes in Lean Mass in Juvenile versus Adult Male F344 Rats
Source: PLoS One. 2015 May 27;10(5):e0125889. doi: 10.1371/journal.pone.0125889 (PMC4446322; doi:10.1371/journal.pone.0125889)
Supplement: S1 Table — Summary of main effects observed following ANOVA at the genus level of taxonomy. These genera were differentially impacted by time, age, and exercise. (DOCX) [file pone.0125889.s001.docx]

**S1 Table. ANOVA main effects summary at the genus level.**

| **Effect** | **PHYLUM** | **Genus** | **Post-FDR p value** | **Direction** |
| --- | --- | --- | --- | --- |
| **Time** | Bacteroides | Bacteroides spp. | 0.027 | Decreased across time |
|  |  | Prevotella spp. | 0.046 | Decreased across time |
|  | Deferribacteres | Mucispirillum spp. | 0.012 | Decreased across time |
|  | Firmicutes | Lactobacillus spp. | 0.032 | Decreased across time |
|  |  | Anaerofustis spp. | 0.048 | Increased across time |
|  |  | Coprococcus spp. | 0.048 | Decreased across time |
|  |  | Peptococcaceae g__ | 0.05 | Decreased across time |
|  |  | Eubacterium spp. | 0.037 | Decreased across time |
|  |  | Allobaculum spp. | 0.012 | Decreased across time |
|  |  | Christensenellaceae g_ | 0.02 | Decreased across time |
|  |  | Clostridiales f_ g_ | 0.02 | Increased across time |
|  | Proteobacteria | Sutterella spp. | 0.046 | Decreased across time |
|  |  | Desulfovibrio spp. | 0.003 | Decreased across time |
| **Age** | Actinobacteria | Bifidobacteria spp. | 0.048 | Increased in juveniles |
|  |  | Adlercreutzia spp. | 0.012 | Increased in adults |
|  | Bacteroidetes | Prevotella spp. | 0.012 | Increased in juveniles |
|  |  | Rikenellaceae g_ | 0.048 | Increased in juveniles |
|  | Firmicutes | Coprococcus spp. | 0.046 | Increased in juveniles |
|  |  | Bacillaceae g_ | 0.012 | Increased in adults |
|  |  | Streptococcus spp. | 0.046 | Increased in adults |
|  |  | Clostridium spp. | 0.032 | Increased in adults |
|  |  | Erysipelotrichaceae g_ | 0.012 | Increased in adults |
| **Exercise** | Euryarchaeota | Methanosphaera spp. | 0.042 | Increased by exercise |
|  | Firmicutes | Turicibacter spp. | 0.02 | Increased by exercise |
|  |  | Dehalobacteriaceae g_ | 0.048 | Increased by exercise |
|  |  | Lactococcus spp. | 0.02 | Increased by exercise |
|  |  | Oscillospira spp. | 0.037 | Decreased by exercise |

Summary of main effects observed following ANOVA at the genus level of taxonomy. These genera were differentially impacted by time, age, and exercise.
